# Foreign Summary

**Published:** 1839-01-19

**Authors:** 


### FOREIGN SUMMARY.

*Dr. Graves on the Cure of Ulcers.*—A case, which deserves a brief notice, is that of a man in the chronic ward, who had old ill-conditioned ulcers on his thighs when admitted. These ulcers constantly discharged a quantity of foul sanies and unhealthy pus, and had remained open for several weeks. Mr. Parr applied an astringent lotion for some days; but as I thought I could cure him much more expeditiously, I ordered the lotion to be discontinued, and had the sores carefully dressed with the beaver or fur plucked from a hat. This was applied with the view of absorbing the discharge, and concreting, so as to form an artificial scab, which tends at once to protect the raw surface and check the formation of fresh matter. The result accorded with my anticipations in this instance, for the ulcers dried up and healed in a few days. I have followed this practice of producing an artificial scab for the last fourteen years, and I can recommend it strongly as applicable to the successful treatment of certain descriptions of chronic sores, and, indeed, of some recent wounds. Let us consider for a moment how the consolidation of detached or divided parts is accomplished. In all your books on surgery you have an account given of a process which is termed adhesion, or adhesive inflammation, which, in itself, is looked upon as a kind of morbid process. It is unnecessary for me to enter into any argument to prove the fallacy of this notion, for Dr. Macartney has established the fact, that what has been termed adhesive inflammation exists no where except in the minds of surgical writers. He has shown that healing is always most rapid and complete where no inflammation exists. He has proved that wounds in which no inflammatory disturbance is going on, heal most readily; that where there is a simple incised wound, or solution of continuity without laceration or bruising, and where proper care is taken to keep the parts in complete apposition, they unite, not by means of inflammation, *but by a process of nutrition.* When parts have been simply divided and then placed in apposition, fluids are poured out, which, coagulating, glue them together and accomplish their union; and there is just as much need of inflammation to complete this process as there is to produce the various parts of the fœtus. It is not, therefore, necessary, for the cure of solutions of continuity, that any inflammation whatever should exist; and Dr. Macartney has conferred a great boon on society by showing that, in such instances, the cure is established by a mere physiological process. We know that in numerous cases where a bullet or needle has been lodged in the flesh, it will go from one part of the body to another, causing solutions of continuity more or less extensive, all of which are repaired without any consequent inflammation. We know, also, that when a simple wound is closed by a clot of blood, it generally heals without inflammatory symptoms. It becomes, therefore, a question in what cases we can imitate this process of nature; and, without entering further into theory, I may observe, that many chronic open sores which refuse to heal even when treated according to the most approved principles of surgery, by means calculated to reduce inflammation or promote granulation and cicatrization by bandages, adhesive plaster, ointments, caustic, &c., will often heal with great rapidity, by covering them with the fur of a beaver hat, carded cotton-wool, or any similar substance, and thus permitting the natural exudation to concrete and form an artificial scab. When this is effected, the edges of the ulcer approximate, and the sore cicatrizes. This process, however, proves successful only when you succeed in making the scab adhere as well over the whole surface as at the edges. But even in case it should fail, it will not do any harm, or prevent you from trying other remedies. The cases in which it is most likely to prove unsuccessful is where there is a copious discharge of ichorous watery fluid, and where much irritation or inflammation exists. Where there is only a copious watery discharge, and no remarkable irritation present, you will often succeed by irritating or scratching the surface of the sore so as to make it bleed. The blood which exudes from the sore concretes readily, and a scab is formed in a very short space of time. By this plan I have repeatedly succeeded in making open sores and old buboes heal which had resisted other means. It is worthy of remark, that an analogous mode of treatment has been found very serviceable in the ulcers and wounds of trees; for the celebrated cement, invented by Dr. Forsyth, and the grafting wax of gardeners, both act on the principle of forming an artificial and intimately adhering scab. In recent wounds, even where considerable injury is inflicted, and where loss of substance has been caused, it is often quite possible to make use of this method; it has been supposed by most, if not by all writers, that bruised and injured parts require suppuration, first for their removal, and secondly for their renewal; but I deny this, and I could appeal to numerous cases where extensive loss of substance was replaced *under a scab* without suppuration, and where bruised parts were removed without suppuration.

The powers of the living tissues and vessels are quite adequate to the deposition of new and the absorption or removal of old matter, without the necessary intervention of suppuration. I have seen a great portion of the fleshy part of the tip of the finger nipped off by the clapping of a door in a steam carriage; I have dressed it with fur of hat; a scab has been formed, and under that scab the bruised parts have been absorbed, and the destroyed parts replaced without the formation of a drop of pus. *As inflammation is a mere accident, not necessarily connected with healing by the first intention, so is suppuration a mere accident, and is not essential to the replacement of loss of substance.—London Med. Gaz.*

---

*Treatment of Typhus Fever.* By Dr. Graves.—The appearance of the patient, on admission, was dorsal decubitus, moaning, unable to move without assistance, her face flushed, skin of a burning heat, and eyes much suffused; her tongue was furred, pulse 130 and weak, and respiration laboured—about 40 in a minute. No dullness on percussion, existed, but loud, sonorous, and sibilous rales were heard all over the chest, particularly in the anterior regions. It was clear, therefore, that our patient had two distinct diseases—spotted fever and bronchitis: the former had commenced with symptoms of unusual violence, and the latter of a formidable nature, engaging a a vast number of the smaller bronchial tubes, and interfering most unfavourably with the breathing. In short, gentlemen, we seemed called upon to treat, in the same individual, two affections of opposite characters—the one inflammatory, and requiring antiphlogistic measures—the other exhibiting a strong tendency to putrescence, and, at the actual stage of her illness, rapidly giving rise to sinking and exhaustion.

Emergencies of this kind require much deliberation, and are calculated to excite strong feelings of embarrassment and doubt in the mind of the practitioner, for he possesses no general rule to direct—no theory to indicate the treatment. Under such circumstances, how happy do we feel if enabled to call to mind some case of a similar description, where we had observed the effects of treatment with anxiety, and noted them with due accuracy; how cheeringly does the light of the past then dispel the obscurity of the present; and how thankfully do we accept the guidance of recorded facts, without whose aid all would be uncertainty and confusion!

Were we, in treating our patient, to direct our attention chiefly to the extensive bronchitis, and prescribe cupping, leeches, blisters, calomel, squills, digitalis, or ipecacuanha? or were we to strive rather against the general than the pectoral ailment? The following considerations seemed capable of affording a solution of these difficulties. In the first place, we had to attach a proper degree of importance to the period of her disease: the spotted fever had already lasted eleven days, after which the symptoms of reaction usually begin to decline, and are followed by a rapid and alarming prostration of strength, requiring the administration of stimulating medicines and a more nutritious food. But here the question occurred, would not nourishment and wine increase the bronchial inflammation, and might they not augment the amount of local disease, by inducing pulmonary congestion, or even pneumonia?

Dr. Stokes, who, during my temporary absence, had the charge of this patient, evinced his usual skill and judgment, and justly regarding the fever the principal disease, he determined to give wine and chicken broth, with a mixture containing carbonate of ammonia. She got one bottle of port wine in the course of the next twenty-four hours; while an attempt was made to relieve the chest by dry cupping, blisters, and small doses of hyoscyamus and ipecacuanha. The same medicines were continued on the succeeding day, but the wine was diminished about one-fourth. Let us now examine this patient, and ascertain what effects have been produced by this treatment, now continued forty-eight hours. You observe, gentlemen, a striking improvement: the fever has a much less threatening aspect, the maculæ have nearly disappeared, the tongue become much cleaner, skin more natural, belly soft, sleep tranquil, and the pulse has nearly fallen to its normal frequency. Such is the result of the treatment, as far as the fever is concerned; but what changes has the bronchitis undergone? Here the effect produced is most satisfactory, for, so far from increasing under the use of wine, moderate nourishment, and carbonate of ammonia, the bronchial inflammation has notably subsided,—the woman's breathing is now comparatively free and calm, and she is less troubled with cough, while the sonorous and sibilous rales are rapidly becoming less distinct. It is our intention to continue the wine and ammonia, but in daily diminishing quantity, and I have no doubt that our patient will soon be able to do without them entirely.*

This case is well deserving a more careful analysis. With respect to the fever, it was evidently accompanied, in its accession and first stage, by active conjestion of the gastro-intestinal and bronchial mucous membrane. Had we been consulted during the rigors, or the first six hours, a bold use of the lancet would have cut short the disease; on the second day, at least during the first half of it, venesection would have been required, but in smaller quantity, and with the effect, not of stopping the fever, but of moderating all the most prominent and distressing symptoms; for the next two days, leeches to the epigastrium and sternum, frequently applied, six at a time, would have powerfully contributed to produce a more natural state of the gastric and bronchial mucous membrane: unhappily the case was neglected before its admission into our ward, and then we were unable to proceed boldly with depletory measures, as the maculæ were very abundant and the fever had arrived at its ninth day. But, here some one will observe, inflammation of a mucous membrane must be treated on the same principles, no matter whether it occurs in the commencement, the *acmé*, or the decline of fever: in every case it must be met by depletory measures, modified, indeed, as to their activity, by the period of the disease and the strength of the patient; *but still depletory they must be;* and in no case are stimulants allowable—in no case wine and nourishment admissible. Gentlemen, there seems to be much that is reasonable in these observations, and they obtain additional confirmation from the testimony of those who assure us, that in persons affected with typhus accompanied with pectoral and abdominal symptoms, such as we witnessed in Mary Lynch, and in whom the disease proves fatal, the post-mortem examination always exhibits indubitable evidence of inflammation affecting the mucous membranes in question; but I must confess that I am more inclined to rely on the effects of remedies as a test of the true nature of diseases, than on the evidence derived from post-mortem examinations. The manner in which a living tissue conducts itself, when acted on by food or medicine, is with me of greater value in determining the nature of a disease affecting that tissue, than any appearances observable in it after death. Suppose that Lynch had died just as we had ordered the wine, broth, and carbonate of ammonia, and suppose her chest and abdomen examined in a few hours after, then I have no doubt that appearances enough would have been detected in the stomach and bronchial tubes to convince you of the existence of mucous inflammation, and you would have been apparently justified in strongly condemning our practice. This I say advisedly, and with what intention? To decry the utility of morbid anatomy? Certainly not. I cannot, indeed, now discuss the general question as to the manner in which morbid anatomy should be prosecuted, in order to render it useful to practical men; but this much I may be permitted to remark, that in the hands of some of its most zealous cultivators, it has degenerated into a system in which the facts discovered have not been arranged and valued according to their obvious bearings, but have been made the foundation of much useless pathological mystification; in truth, it is my fixed opinion, founded on no small opportunities of observation, that in fever especially, much error and uncertainty still attend the interpretation (if that expression may be used) of *post-mortem* appearances, and consequently great mischief has resulted from rules of practice derived rather from the dead-room than from the clinical ward.

But to return to our patient. Her case teaches you how, in the progress of typhus fever, the state of the mucous membrane of the stomach and bowels alters, and how its vital energies vary as the disease advances; for we see wine required,—we see it borne,—nay, useful, not only to the system at large, but to the alimentary mucous membrane in particular, on the eleventh day of fever, in a case where it would have been injurious, perhaps destructive, at an earlier stage; and mark, that it was thus useful, *even although all the symptoms which might be justly considered as contraindicating its exhibition had not entirely disappeared!* We learn from this that our feeble powers are incapable of appreciating the meaning, the intensity, or even, in every case, the identity of particular symptoms. We must, therefore, never sacrifice general views to indi-

* This patient perfectly recovered, to the no small astonishment of two foreign physicians who were somewhat tinged with *Broussaism*.

vidual indications, and, in fever especially, *we must study the whole man, and not any particular organ.—Ib.*

---

*Chemical Examination of the Liquor Amnii.* By G. O. Rees, M. D.—This secretion has been examined by several continental chemists, with a great diversity of results, not only as to the quantities, but likewise the quality of its constituents. Buniva and Vauquelin make its specific gravity 1004; and its solid contents 11 to 12 parts in 1000. Frommhertz's experiments differ from the late observations of Dr. Vogt, of Bern; who attempts to explain the discrepancy, by supposing that the fluid, as examined by Frommhertz, was not obtained pure.

In a paper on Albuminous Fluids, by Dr. Bostock, published in the fourth volume of the "Medico-Chirurgical Transactions," the solid contents of a specimen of liquor amnii is given as 16.6 in 1000 parts; which would make the specific gravity of the fluid to be about 1008.6. This observation is in close accordance with that of Dr. Rees; the four specimens which he examined varying in specific gravity between 1007 and 1008.6. The analyses were performed on specimens of liquor amnii with which no extraneous matter was admixed, the liquor being drawn off either by a canula or a female catheter.

C. W. has been delivered of three children. In the two first labours, the perforator was employed to effect delivery. The third labour was brought on at 7½ months; the child born dead. On examination, the brim and outlet of the pelvis were found to be contracted; labour was, therefore, induced at 7½ months, by passing a female catheter into the os uteri, and drawing off the liquor amnii. Labour pains came on twenty-four hours after the operation, and in forty-one hours a female child was born alive; it lived only half an hour.

*Examination of Liquor Amnii.*

Strongly alkaline—Sp. grav. 1008.6.

Contained in 1000 parts:

| | |
|---|---|
| Water, | 983.4 |
| Albumen, (traces of fatty matter,) | 5.9 |
| Albuminate of soda, } Chloride of sodium, } | 6.1 |
| Animal extractive soluble in water and alcohol, urea, chloride of sodium, | 4.6 |
| Traces of alkaline sulphate. | |

The examination of a second specimen gave in 1000 parts:

| | |
|---|---|
| Water, | 984.98 |
| Albumen, (traces of fatty matter,) | 1.80 |
| Extract soluble in water—Salts, 2.80; organic matter, principally albumen, from albuminate of soda, 3.22, | 6.02 |
| Extract soluble in water and alcohol—Salts, 2.80; organic matter principally lactic acid and urea, 4.4, | 7.20 |

The third specimen examined was taken from the same patient as specimen 1. The liquor was abstracted in the same manner, and at the same period of utero-gestation (7½ months.) The fœtus was a female, and still-born. Contained in 1000 parts:

| | |
|---|---|
| Water, | 986.8 |
| Albumen, (traces of fatty matter,) | 3.2 |
| Aqueous extract—Salts, 3.2; organic matter, viz., albumen, from albuminate of soda, 1.2, | 4.4 |
| Alcoholic extract—Salts, 3.8; organic matter, viz., lactic acid and urea, 1.8, | 5.6 |

In the fourth specimen were:

| | |
|---|---|
| Water, | 986.8 |
| Albumen, (traces of fatty matter,) | 2.4 |
| Aqueous extract—Salts, 4.2; organic matter, viz., albumen, from albuminate of soda, 2.0, | 6.2 |
| Alcoholic extract—Salts, 3.0; organic matter, viz., lactic acid and urea, 1.6, | 4.6 |

The fatty matter procured from the liquor amnii differs from that of the blood, in not assuming a deep purple tint when digested in strong sulphuric acid.

The salts, both of the aqueous and alcoholic extracts, consisted of chloride of sodium and carbonate of soda, with minute traces of an alkaline sulphate and phosphate. In the salts of the aqueous extractive, the carbonate resulted from the incineration of an albuminate; and in the alcoholic extractive, from the decomposition of a lactate, by the same operation.

The salts obtained from the aqueous extractive in two analyses were not entirely soluble in water. The insoluble matter, on examination, proved to be phosphate of lime, which must either have been held in solution with the albuminate of soda, or have resulted from the decomposition of an alkaline phosphate at a red heat; some soluble earthy salt being present to effect such decomposition.

Urea was proved to exist in the liquor amnii, by its characteristic crystallization with nitric acid, and the re-actions of its nitrate. A further evidence was obtained in the analysis of specimen 3 by the crystallization of chloride of sodium in octohedra, from the alcoholic extract, when allowed to evaporate spontaneously. Specimen 3 contained urea in greater proportion than the other specimens.

The albumen procured from the liquor amnii yields a trace of oxide of iron and earthy phosphate, on being incinerated.

The flocculi which are observed floating in the liquors are composed of caseous matter, containing cholesterine.

On examining the analyses, it will be observed that the liquor amnii varies greatly in proportional constitution in different individuals, at the same period of utero-gestation, which shows that, like, perhaps, all the secretions of the body, it is affected by the temperament and diathesis of the mother. The specific gravity of the secretion, however, varies but little in the four specimens, which is possibly a precaution on the part of nature to preserve a medium of fixed power, to oppose the motions of the fœtus in utero.

*Lancet.*

